# Psychometric Testing of the CHAMPS Questionnaire in French Canadians with COPD

**DOI:** 10.1155/2019/2185207

**Published:** 2019-09-17

**Authors:** Susanne Mak, Jean Bourbeau, Nancy E. Mayo, Sharon Wood-Dauphinee, Judith E. Soicher

**Affiliations:** ^1^McGill University, School of Physical & Occupational Therapy, Montréal H3G 1Y5, Québec, Canada; ^2^McGill University Health Centre Research Institute, Montréal H4A 3S5, Québec, Canada

## Abstract

Physical activity is an important health behaviour in reducing morbidity and mortality in individuals with chronic obstructive pulmonary disease (COPD). Accurate measurement of the characteristics of physical activity is essential to understanding the impact of COPD on physical activity. In a previous article, we reported on the cross-cultural adaptation of the Community Healthy Activities Model Program for Seniors (CHAMPS) questionnaire to produce a Canadian French version. The CHAMPS yields four summary scores: two caloric expenditure scores (moderate-intensity activities and all activities) and two frequency scores (moderate-intensity activities and all activities). The objective of this study was to evaluate test-retest reliability and convergent construct validity, in both English and French versions of the CHAMPS, in individuals with COPD. Test-retest reliability was assessed by administering the CHAMPS at two visits (2-3 weeks apart), to 19 English-speaking and 18 French-speaking participants. Validity was assessed in 56 English-speaking and 74 French-speaking participants, who completed the CHAMPS, Short Form- (SF-) 36, and St. George's Respiratory Questionnaire (SGRQ) at a single visit. Results from reliability testing indicated that intraclass correlation coefficients (ICCs) generally met the threshold for good reliability (ICC > 0.6), with frequency scores showing greater stability than caloric expenditure scores. Validity testing yielded moderate correlations (*r* = 0.4-0.5) of the CHAMPS with the SF-36 domains and summary score capturing constructs of physical function, and with the SGRQ activity domain and total score. CHAMPS frequency scores for moderate-intensity activities correlated more strongly than other scores, with physical aspects of the SF-36 and SGRQ. The English and French versions of the CHAMPS did not show any substantial differences in reliability (frequency scores) or validity (frequency and caloric expenditure scores). Findings from this study support the reliability and validity of the CHAMPS. In particular, frequency scores for moderate-intensity activities can provide useful information on physical activity levels in individuals with COPD. This trial is registered with NCT00169897. ISRCTN registration number: IRSCTN32824512.

## 1. Introduction

Chronic obstructive pulmonary disease (COPD) is among the leading causes of mortality and morbidity worldwide [1]. Physical activity is an important health behaviour in reducing morbidity and mortality in COPD patients, and a lower level of physical activity early in the disease process has been associated with hospital readmission [2]. However, COPD patients often experience a worsening of their pulmonary function and health state and are not able to optimally participate in physical activity [3, 4].

Accurate measurement of the characteristics of physical activity (e.g., type, duration, and intensity) is essential to understanding the impact of COPD on physical activity [5]. Common physical activity measures include wearable devices such as pedometers, accelerometers, heart rate monitors, and multisystem sensors [6]. While these devices provide numerical estimates of physical activity, they may be expensive, require specific expertise for data analysis, be susceptible to electro-magnetic interference, or function only in specific activity conditions [6, 7]. Questionnaires can also estimate the types and characteristics of physical activities in which patients engage. Compared with wearable devices, questionnaires are less costly, have less respondent burden, and data analysis is more straightforward [6, 7].

The Community Healthy Adults Model Program for Seniors (CHAMPS) questionnaire is a 41-item, self-report questionnaire in which the respondent reports the type and frequency of physical activities carried out in a typical week during the past month. The CHAMPS yields four summary scores: two caloric expenditure scores (moderate-intensity activities and all activities) and two frequency scores (moderate-intensity activities and all activities). In a previous article [8], we reported on forward translation, backward translation, pretesting, and cognitive debriefing, which produced a Canadian French version of the CHAMPS ready for psychometric testing. Reliability is a psychometric property that indicates the stability or reproducibility of a measure [9, 10], allowing a clinician or researcher to interpret scores with confidence and make decisions accordingly. Because the CHAMPS is self-administered and does not involve external raters, test-retest is the appropriate method of reliability testing. An intraclass correlation coefficient (ICC) of 0.70 or higher is considered an acceptable level of reliability for research purposes [10], while a lower value is acceptable for clinical use. Validity is the ability of a questionnaire or instrument to measure the concept that it intends to measure, and is composed of face, content, construct, and criterion validity [11, 12]. Construct validity is commonly used to ascertain the agreement or correlation between instruments that assess a similar construct [11]. It can also be called convergent construct validity when measures are expected to agree or converge [11]. Cohen's criteria can be used to interpret the magnitude of correlation as small (0.2 ≤ *r* < 0.5), moderate (0.5 ≤ *r* ≤ 0.8), or large (*r* ≥ 0.8) [13].

Reliability and validity of the original English CHAMPS questionnaire were previously evaluated in a large sample of 249 older Americans, 10% of whom reported a chronic respiratory condition [14]. In this study, Stewart et al. found acceptable levels of reliability (ICC = 0.58–0.67) [14]. For construct validity, comparison of the CHAMPS to other health-related measures (e.g., 6-minute walk test, lower body functioning, and self-reported physical functioning) yielded correlations of small magnitude (*r* = 0.20–0.30) [14]. In other studies, similar correlations were observed for construct validity [15, 16].

While past research provides evidence of the CHAMPS' reliability and construct validity, these studies were conducted in a general, older adult population. Because psychometric properties are specific to the patient population, language, and context in which a measure is used, further psychometric testing is required in English and French linguistic subgroups of the COPD population. The overall aim of our study was therefore to evaluate test-retest reliability and convergent construct validity, in both English and French versions of the CHAMPS, in individuals with COPD.

Specific objectives were as follows:

- To estimate the test-retest reliability of the original (English) and the Canadian French CHAMPS. We hypothesized moderate to good test-retest reliability (ICC > 0.60) for all summary measures (caloric expenditure and frequency) in both the English and French versions.
- To estimate the convergent construct validity of the original (English) and the Canadian French CHAMPS. We hypothesized moderate correlations (*r* = 0.4-0.5) of the CHAMPS summary measures with the Short-Form Health Survey (SF-36) and the St. George's Respiratory Questionnaire (SGRQ), both measures of health-related quality of life. Specifically, we expected higher correlations of the CHAMPS with the SF-36 domains and component summary scores capturing constructs of physical function, and with the SGRQ activity domain and total score.

The hypothesized values for reliability and validity were based on results from previous studies [14, 15] and established cut-off values [10, 11, 13].

## 2. Methods

### 2.1. Study Design

Assessment of test-retest reliability used a longitudinal design in which the CHAMPS was administered at visit 1 and repeated 2-3 weeks later at visit 2. Construct validity was assessed in a separate group of participants using a cross-sectional design, in which the CHAMPS, SF-36, and SGRQ were administered at a single time point [17]. Participants completed either French- or English-language questionnaires, according to their reported first language.

### 2.2. Study Sample

#### 2.2.1. Test-Retest Reliability

Participants were recruited from the COPD clinic of the Montreal Chest Institute (McGill University Health Centre). Inclusion criteria were (1) age ≥40 years; (2) current or previous smoker with a smoking history of at least 10 American pack-years; (3) forced expiratory volume in 1 second (FEV_1_) after bronchodilator <70% of the predicted normal value and a ratio of forced expiratory volume in 1 second to forced vital capacity (FEV_1_/FVC) <70%; (4) ability to read and understand English or French; and (5) disease stability for two weeks prior to enrolment, defined as no important change in respiratory medications, symptoms, health-related quality of life, or spirometry.

Exclusion criteria were (1) a primary diagnosis of asthma; (2) personal or professional obligations causing changes in physical activity habits during the two-week study period (which may impact on test-retest reliability); or (3) a terminal illness, dementia, or uncontrolled mental health condition. At visit 2, participants were asked about changes in physical activity habits and respiratory symptoms since visit 1. Participants remained eligible to complete visit 2, as long as they reported no substantial change in physical activity habits or respiratory symptoms (cough, sputum, and shortness of breath).

A sample size of 20 participants for each version of the CHAMPS was targeted for reliability testing [18]. Ethical approval was obtained from the Research Ethics Board of the McGill University Health Centre, and participants provided written informed consent.

#### 2.2.2. Convergent Construct Validity

The study sample for validity testing consisted of a subgroup of participants enrolled in a multicentre randomized controlled trial (RCT) comparing the effectiveness of outpatient, hospital-based versus self-monitored, home-based exercise training [17]. Participants were included in the validity testing if their data was complete, and they had agreed to the use of their data in future analyses. RCT data collected at the initial (baseline) visit was used to compare the CHAMPS with other measures.

Inclusion criteria consisted of those listed earlier for test-retest reliability, as well as the following additional criteria required in the RCT: disease stability for four weeks prior to study start, Medical Research Council (MRC) Dyspnea Scale score of at least two out of five (level 2 corresponds to shortness of breath walking up a slight hill), and no history of congestive heart failure [17].

Data were obtained from five of the ten sites participating in the RCT [17]: Queen Elizabeth II Health Sciences Centre (Halifax, NS); Hôpital Laval (Quebec City, QC); Montreal Chest Institute of the McGill University Health Centre (Montreal, QC); Mount Sinai Hospital Centre (Montreal, QC); and St. Paul's Hospital (Vancouver, BC). Ethical approval was obtained from the research ethics board of all sites, and participants provided written informed consent.

### 2.3. Study Measures

Participants' baseline sociodemographic and clinical information was collected, including age, sex, body mass index, dyspnea, FEV_1_, FEV_1_ (% predicted), FEV_1_/FVC, marital status, smoking status, and number of comorbid conditions. Dyspnea was evaluated using the Medical Research Council Dyspnea Scale (MRC), scored from 1 to 5.

The following self-administered questionnaires were explained using standardized instructions and then were completed by participants. The principal investigator (SM) or a research assistant was present to answer questions or provide clarification as needed. The questionnaires were administered in the same order to all participants.

#### 2.3.1. CHAMPS

The CHAMPS consists of 41 items that assess the frequency and duration of a specific physical activity in a typical week during the past month. An example questionnaire item is: “In a typical week during the past 4 weeks, did you walk leisurely for exercise or pleasure?” Frequency is reported as the number of times the activity was performed during a typical week. For duration, the participant estimates the total hours per week spent doing the activity and chooses one of six response options, ranging from less than one hour to 9 or more hours. Duration and METs (metabolic equivalents) are used to calculate the energy expenditure required for a given activity. One MET is 3.5 ml of oxygen per kilogram of body weight per minute and represents the energy expenditure of a person at rest [19].

Summary measures of caloric expenditure and frequency are generated for all items/activities corresponding to any MET value and for items/activities of at least moderate intensity (MET value ≥3.0) [14]. Therefore, four separate scores can be calculated from the CHAMPS questionnaire.

#### 2.3.2. Short Form- (SF-) 36

The SF-36, a generic health-related quality of life questionnaire, consists of 36 items divided among eight domains: physical functioning, social functioning, physical health, emotional health, pain, vitality, mental health, and general perception of health [20, 21]. Scores from 0 to 100 are calculated for each domain, as well as physical and mental component summary scores [20, 21]. The SF-36 has been translated into Canadian French and has demonstrated evidence of internal consistency, test-retest reliability, and construct validity in a COPD population [20, 22].

#### 2.3.3. St. George's Respiratory Questionnaire (SGRQ)

The SGRQ, a disease-specific, health-related quality of life questionnaire, consists of 76 items divided into three domains: symptoms, activity, and impact. Scores are calculated out of 100 for each domain and for the questionnaire as a whole, with higher scores indicating poorer health. The SGRQ has been translated into French and has demonstrated strong psychometric properties in the French-Canadian COPD population [23–25].

### 2.4. Statistical Analysis

To assess test-retest reliability of the CHAMPS, ICCs were calculated for caloric expenditure and frequency, based on data from the first and second visits. The ICCs and 95% confidence intervals were estimated using repeated measures ANOVA [10]. ICCs were then compared to the hypothesized values.

For convergent construct validity, correlation coefficients were calculated to determine the association of CHAMPS scores with: (i) the SF-36 domain and component summary scores and (ii) the SGRQ domain and total scores. Due to the non-normal distribution of CHAMPS scores, Spearman's correlation coefficients were calculated and then compared to the hypothesized values. Data analysis was performed using SAS® version 9.1.2 (SAS Institute Inc., CA, USA).

## 3. Results

### 3.1. Participant Characteristics

In the reliability testing, 52 participants consented to participate and completed visit 1. Three participants withdrew from the study and 12 participants did not complete visit 2, either due to inability to attend a return visit or due to a reported change in disease status or physical activity. Therefore, the final sample for reliability testing consisted of 19 English-speaking and 18 French-speaking participants.

The English- and French-speaking groups were typical of the COPD population, but differed with respect to several sociodemographic and clinical characteristics (see Table 1). There were more males than females in both participant groups, with a larger proportion of males in the English-speaking group (79%) than in the French-speaking group (56%). A larger proportion of French-speaking participants reported severe dyspnea (50%) compared with the English-speaking group (37%). More French-speaking participants self-identified as “never/formerly married” (67% versus 42% in the English-speaking group) and reported one or more comorbid conditions (78% versus 58% in the English-speaking group).

In the validity testing, baseline data from 56 English-speaking participants and 74 French-speaking participants were obtained from a multicentre RCT [17]. Examination of the sociodemographic and clinical characteristics demonstrated that the English- and French-speaking participants were similar in terms of age, body mass index (BMI), FEV_1_, FEV_1_/FVC (%), FEV_1_ (% predicted), marital status, and smoking status (see Table 2). A greater proportion of English-speaking participants reported severe dyspnea (32% versus 19% in the French-speaking group) and one or more comorbid conditions (67% versus 46% in the French-speaking group).

### 3.2. Test-Retest Reliability

For each of the four CHAMPS scores, Tables 3 and 4 present the mean and median scores at visits 1 and 2, as well as the ICCs and 95% confidence intervals. Among English-speaking participants (Table 3), ICCs of 0.47 (all activities) and 0.60 (moderate-intensity activities (MET value ≥3.0)) were obtained for caloric expenditure. ICCs of 0.65 (all activities) and 0.61 (moderate-intensity activities) were estimated for frequency scores. Among French-speaking participants (Table 4), ICCs of 0.70 (all activities) and 0.14 (moderate-intensity activities) were obtained for caloric expenditure. ICCs of 0.61 (all activities) and 0.68 (moderate-intensity activities) were estimated for frequency.

### 3.3. Convergent Construct Validity

#### 3.3.1. CHAMPS Caloric Expenditure Scores

Correlations between CHAMPS caloric expenditure and SF-36 scores ranged in magnitude from 0.007 to 0.4 (English) and from 0.04 to 0.5 (French) (Table 5). The following correlations were statistically significant (95% CI does not include zero; *p* < 0.05) and met the threshold for moderate correlation (*r* ≥ 0.4), defined a priori in our study objectives: CHAMPS caloric expenditure (moderate-intensity activities) with SF-36 physical component summary score (*r* = 0.4) in English-speaking participants and CHAMPS caloric expenditure (moderate-intensity activities) with SF-36 physical functioning (*r* = 0.5) in French-speaking participants. Two other correlations, which were not included in our study hypotheses, also reached the threshold for moderate correlation: CHAMPS caloric expenditure (moderate-intensity activities) with SF-36 social functioning (*r* = 0.4) in French-speaking participants and with SF-36 vitality (*r* = 0.4) in English-speaking participants.

Correlations between CHAMPS caloric expenditure and SGRQ scores ranged in magnitude from 0.02 to 0.5 in both English and French participant groups (Table 5). Correlations between CHAMPS caloric expenditure (moderate-intensity activities) and SGRQ activity (*r* = −0.5) and total scores (*r* = −0.4) were statistically significant and reached the threshold for moderate correlation in both participant groups. Note that negative correlations are due to the scoring of the SGRQ, in which lower scores represent better health.

#### 3.3.2. CHAMPS Frequency Scores

Correlations between CHAMPS frequency and SF-36 domains ranged in magnitude from 0.06 to 0.4 (English) and from 0.02 to 0.5 (French) (Table 6). The following correlations were statistically significant and met the threshold for moderate correlation (*r* ≥ 0.4): CHAMPS frequency (moderate-intensity activities) with SF-36 physical role limitations (*r* = 0.4) and physical component summary score (*r* = 0.4) in English-speaking participants and CHAMPS frequency (moderate-intensity activities) with SF-36 physical functioning (*r* = 0.5) in the French-speaking group. Three other correlations, which were not included in our study hypotheses, also reached the threshold for moderate correlation: CHAMPS caloric expenditure (moderate-intensity activities) with SF-36 general health, social functioning, and vitality (*r* = 0.4) in English-speaking participants.

Correlations between CHAMPS frequency and SGRQ domains ranged in magnitude from 0.1 to 0.5 (English) and from 0.09 to 0.4 (French) (Table 6). The following correlations were statistically significant and met the threshold for moderate correlation (*r* ≥ 0.4): CHAMPS frequency (all activities) with SGRQ activity (*r* = −0.4) in the English-speaking group; CHAMPS frequency (moderate-intensity activities) with SGRQ activity (*r* = −0.5) and total scores (*r* = −0.4) in the English-speaking group; and CHAMPS frequency (moderate-intensity activities) with SGRQ activity (*r* = −0.4) and total scores (*r* = −0.4) in the French-speaking group. Correlations between CHAMPS (moderate-intensity activities) and SGRQ impact, although not included in our study hypotheses, reached the threshold for moderate correlation in both linguistic groups (*r* = 0.4).

## 4. Discussion

This study investigated the test-retest reliability and convergent construct validity of both English and French versions of the CHAMPS questionnaire in individuals with COPD. ICCs generally met or exceeded the threshold for good reliability (>0.6). Validity testing yielded moderate correlations (0.4-0.5) of the CHAMPS with the SF-36 domains capturing constructs of physical function and physical component summary score and with the SGRQ activity domain and total score. The English and French versions of the CHAMPS did not show any substantial differences in reliability (frequency scores) or validity (frequency and caloric expenditure scores). In the following discussion, we interpret our results with respect to the study objectives and hypotheses, findings from previous studies of psychometric properties, and overall trends, strengths, and limitations observed in the current study.

### 4.1. Test-Retest Reliability

In our study, we hypothesized moderate test-retest reliability (ICC > 0.60) for both summary measures (caloric expenditure and frequency) in both the English and French versions of the CHAMPS. For CHAMPS caloric expenditure, this threshold was reached, with the exception of scores for all activities in the English-speaking group (ICC = 0.47) and moderate-intensity activities in the French-speaking group (ICC = 0.14). Large differences in caloric expenditure scores between visits 1 and 2 were observed in seven of the 18 French-speaking participants, despite reports of slight or no changes in physical activity habits or respiratory symptoms. This large between-visit difference in scores, noted in over a third of the French-speaking sample, led to a lower ICC for moderate-intensity activities. This observation was not seen in ICCs for caloric expenditure (all activities) and frequency.

In both linguistic groups, all ICCs for frequency exceeded the threshold value of *r* = 0.6 and were more consistent than those observed for caloric expenditure (range 0.61 to 0.68). These values were similar to findings from a previous study in a large sample of older adults (*n* = 147) [14]. In this study, Stewart et al. reported ICCs over a 2-week interval of 0.66 (all activities) and 0.67 (moderate-intensity activities) for caloric expenditure, and ICCs of 0.62 (all activities) and 0.58 (moderate-intensity activities) for frequency [14]. In another large study of older adults (*n* = 167), Cyarto et al. obtained ICCs over a 1-week interval of 0.79 (all activities) and 0.81 (moderate-intensity activities) for frequency [16], however did not report ICCs for caloric expenditure. The authors suggested that the shorter 1-week interval between visits may have led to more stable patient self-reports of physical activity and therefore higher ICCs than in Stewart's study.

### 4.2. Convergent Construct Validity

In our study, we hypothesized moderate correlations (*r* = 0.4-0.5) of the CHAMPS summary scores with the SF-36 and the SGRQ, specifically for domains capturing physical function. Most correlations between the CHAMPS and SF-36 physical domains (physical role limitations, physical functioning, and physical component summary) were small in magnitude and did not reach the threshold for moderate correlation. Nonetheless, our observed correlations for both caloric expenditure and frequency were similar to those observed in past studies. Stewart et al. obtained correlations of 0.27 (all activities) and 0.3 (moderate-intensity activities) between caloric expenditure and the SF-36 physical functioning domain [14], while Harada et al. reported correlations of 0.39 (all activities) and 0.41 (moderate-intensity activities), also in older adults (*n* = 87) [15]. For frequency, Stewart et al. reported correlations of 0.23 (all activities) and 0.3 (moderate-intensity activities) with the SF-36 physical functioning domain [14].

In contrast to the SF-36 physical domains, the correlations between the CHAMPS and the SF-36 emotional and pain domains (mental health, role limitations-emotional, mental component summary, and bodily pain) fell below our threshold for moderate correlation (range 0.02 to 0.3). These lower correlations were as expected, given that the focus of the CHAMPS questionnaire is on physical activity and functioning. Correlations between the CHAMPS and the remaining SF-36 domains (general health, social functioning, and vitality) were generally lower than correlations with SF-36 physical domains, but higher than those with SF-36 emotional and pain domains. This finding is not surprising, as these remaining domains capture broad constructs that reflect both physical and emotional aspects of health-related quality of life.

As hypothesized, in both linguistic groups, moderate correlations were attained between the CHAMPS summary scores for moderate-intensity activities and the SGRQ activity and total scores. Correlations between CHAMPS scores for all activities and the SGRQ activity and total scores were smaller in magnitude and only one (frequency with SGRQ activity in the English group) met the threshold for moderate correlation. Although not included in our hypotheses, correlations between frequency (moderate-intensity activities) and the SGRQ impact domain reached the threshold for moderate correlation in both linguistic groups (*r* = −0.4). This correlation may be explained by several questions contributing to the SGRQ impact score, which capture aspects of physical activity similar to items on the CHAMPS. For example, SGRQ question #16 asks about activities requiring a medium to high level of exertion (e.g., playing sports or games, leaving the house to go shopping, and doing housework) [26].

Correlations between the CHAMPS summary measures and the SGRQ symptoms score did not attain our threshold for moderate correlation. SGRQ symptoms questions ask about the frequency and severity of coughing, sputum, shortness of breath, and wheezing [26]. Thus, given that the content of the SGRQ symptoms questions is not reflected in the CHAMPS, the observed correlations are lower in magnitude than those with the SGRQ activity and impact domains, as well as the total score. To our knowledge, this study is the first to examine the relationship between the CHAMPS and the SGRQ.

Through the numerous comparisons carried out in the validity portion of this study, several overall trends emerged, which can further inform our understanding of the CHAMPS questionnaire. First, CHAMPS summary scores for moderate-intensity activities demonstrated stronger correlations with SF-36 and SGRQ physical domains, than did CHAMPS summary scores for all activities. This trend likely reflects the fact that moderate-intensity activities scores only include items/activities requiring a moderate to high level of physical exertion and functioning (e.g., jogging/running and doing aerobics). All activities scores include moderate-intensity activities, as well as items requiring a lower level of exertion, such as using a computer or playing cards, bingo, or board games.

A second trend was that correlations between CHAMPS frequency scores and physical aspects of HRQL were higher than those observed for CHAMPS caloric expenditure scores. This finding may be explained by the fact that the frequency of an activity during the past week can be recalled more accurately than its duration. Furthermore, the frequency score is a simple sum of frequencies for all items, while caloric expenditure is a calculated value based on the responses for duration, making it more prone to distortion if the duration reported is not exact.

A final trend in the validity testing was that correlations between the CHAMPS and the SGRQ were higher than with the SF-36. This can be explained by the SGRQ being a disease-specific measure, which was developed for a COPD population, and therefore better captures aspects of physical function that are most relevant to these individuals.

### 4.3. Study Strengths and Limitations

A strength of our study was the design, which used two separate linguistic groups, allowing reliability and validity testing in both English and French versions of the CHAMPS. Additionally, we drew from previous research and established criteria for psychometric testing, to develop objectives and hypotheses a priori.

Although sample sizes were close to the recommended values of 20 for reliability and 80 for validity [18] in three of the four subsamples, larger samples would have led to improved precision in statistical estimates. In the reliability testing, our sample size was smaller than anticipated, with 19 English-speaking and 18 French-speaking participants. In other similar studies [14, 16], sample sizes for reliability were considerably larger than in our study, resulting in greater magnitude and precision of reliability estimates. In validity testing, the greater number of correlations reaching the threshold of moderate correlation for the French CHAMPS was likely due to the larger sample size of the French-speaking participant group (*n* = 74). Sub-samples of a similarly large magnitude in other portions of the study would have led to clearer trends in the results for reliability and validity, as well as more accurate comparisons between French and English versions of the CHAMPS.

Matching was not carried out between linguistic groups, and therefore these groups differed in several characteristics. In the reliability testing, the higher proportion of French participants with severe dyspnea and comorbidities may have led to greater fluctuation in symptoms and physical activity, resulting in a larger between-visit difference in caloric expenditure and a lower ICC (moderate-intensity activities). Differences in gender between linguistic groups were less likely to influence ICC values as reliability reflects the stability of the CHAMPS scores within each individual at two time points.

In validity testing, the larger proportion of English participants with severe dyspnea (32% vs 19% French) and comorbidities (67% vs 46% French) suggest greater disease severity in the English group. While these differences should not substantially affect the CHAMPS' association with measures assessing similar constructs, subgroup analysis may have provided further insights. The sample sizes in this study, however, were not large enough to properly evaluate the CHAMPS' validity according to disease severity.

In the reliability testing, all participants completed the second visit. A limitation, however, was that some participants completed the second visit three weeks, instead of two weeks, after the first visit (*n* = 8 in the French-speaking group, *n* = 3 in the English-speaking group). This prolonged retest interval may have contributed to more pronounced between-visit differences in physical activity routines, and therefore a lower ICC for moderately intense physical activities in the French-speaking group. Although similar or longer retest intervals have been used in previous studies [14, 15], these studies mostly included healthy older adults. Given the fluctuation in COPD symptoms, the use of a shorter retest interval in our study (i.e., one week) may have resulted in greater stability in physical activity levels between visits 1 and 2. Using a 2-week time interval, however, was less disruptive to participants' schedules, thereby facilitating recruitment and retention.

Finally, in the validity testing, questionnaires were administered in the same order to all participants, in order to adhere to the procedures of the RCT. This may have introduced bias, if participants' responses to questionnaire items were influenced by the order of questionnaires.

Overall, our findings from reliability and validity testing support the use of the CHAMPS to assess physical activity in individuals with COPD. Due to its strong correlation with physical aspects of health-related quality of life, the CHAMPS frequency score for moderate-intensity activities may be particularly useful to assess physical activity in both clinical and research settings. Although duration of activities and caloric expenditure scores can provide complementary information for individual patient care in a clinical setting, we would recommend further psychometric testing before using caloric expenditure scores as an outcome measure in research.

## 5. Conclusion

The CHAMPS is a reliable and valid measure of physical activity in individuals with COPD. The French-language CHAMPS frequency scores demonstrated similar reliability and convergent construct validity to the original English-language version, thereby providing a useful measure of physical activity for French Canadians with COPD. In both linguistic versions of the CHAMPS, a better understanding is needed of the psychometric properties of the caloric expenditure scores, before they can be used with confidence. Future research should focus on testing other types of validity and responsiveness of the CHAMPS.

## Figures and Tables

**Table 1:** Sociodemographic and clinical characteristics of participants: test-retest reliability.

Characteristic	English-speaking participants (*N* = 19)		French-speaking participants (*N* = 18)	
	Mean (SD)	*N* (%)	Mean (SD)	*N* (%)
*Age*	71 (7)		71 (9)	
*Sex*				
Female		4 (21)		8 (44)
Male		15 (79)		10 (56)
*Body mass index* ^*∗*^	25 (6)		25 (3)	
*Dyspnea (MRC)*				
Mild-moderate (1–3)		12 (63)		9 (50)
Severe (4-5)		7 (37)		9 (50)
*FEV*_*1*_ *(L)*	0.98 (0.48)		1.0 (0.51)	
*FEV*_*1*_ */FVC (%)*	44 (13)		44 (14)	
*FEV*_*1*_ *(% predicted)*	38 (16)		42 (15)	
*Marital status*				
Never married or formerly married		8 (42)		12 (67)
Married or common law		11 (58)		6 (33)
*Smoking status*				
Ex-smoker		17 (90)		17 (94)
Smoker		2 (10)		1 (6)
*Number of comorbid conditions*				
0		8 (42)		4 (22)
1		10 (53)		8 (45)
2-3		1 (5)		6 (33)

^*∗*^Body mass index (BMI) is defined as weight (kg) divided by height (m) squared.

**Table 2:** Sociodemographic and clinical characteristics of participants: construct validity.

Characteristic	English-speaking participants (*N* = 56)		French-speaking participants (*N* = 74)	
	Mean (SD)	*N* (%)	Mean (SD)	*N* (%)
*Age*	66 (10)		65 (8)	
*Sex*				
Female		25 (45)		46 (38)
Male		31 (55)		28 (62)
*Body mass index* ^*∗*^	29 (6)		27 (6)	
*Dyspnea (MRC)*				
Mild-moderate (1–3)		38 (68)		60 (81)
Severe (4-5)		18 (32)		14 (19)
*FEV*_*1*_ *(L)*	1.06 (0.43)		1.09 (0.41)	
*FEV*_*1*_ */FVC (%)*	46 (14)		41 (11)	
*FEV*_*1*_ *(% predicted)*	46 (14)		45 (13)	
*Marital status*				
Never married or formerly married		27 (48)		40 (54)
Married or common law		29 (52)		34 (46)
*Smoking status*				
Ex-smoker		46 (82)		59 (80)
Smoker		10 (18)		15 (20)
*Number of comorbid conditions*				
0		19 (34)		40 (54)
1		30 (54)		22 (30)
2-3		7 (13)		12 (16)

^*∗*^Body mass index (BMI) is defined as weight (kg) divided by height (m) squared.

**Table 3:** Descriptive statistics and reliability coefficients (ICC) of the CHAMPS in English-speaking participants (*N* = 19).

		Visit 1		Visit 2		ICC	95% CI
CHAMPS^*∗*^ score		Mean (SD)	Median (IQR^†^)	Mean (SD)	Median (IQR)		
Caloric expenditure^‡^ (kcal/week)	(1) All activities	2513 (1453)	2732 (1256)	2162 (1459)	1626 (2423)	0.47	0.21–0.71
	(2) Moderate-intensity activities	1011 (932)	735 (1473)	907 (1026)	452 (1518)	0.60	0.38–0.80
Frequency (times/week)^§^	(3) All activities	18 (14)	16 (13)	18 (11)	18 (19)	0.65	0.44–0.83
	(4) Moderate-intensity activities	6.6 (6.1)	6 (4)	6.6 (6.8)	4 (11)	0.61	0.39–0.80

^*∗*^CHAMPS = Community healthy activities model program for seniors. ^†^IQR = interquartile range. ^‡^Caloric expenditure scores include (1) all activities defined as the total kilocalories expended during activities of any intensity during a typical week and (2) moderate-intensity defined as the total kilocalories expended during activities of at least moderate intensity (MET value ≥ 3.0) during a typical week. ^§^Frequency scores include (3) all activities defined as the number of activities of any intensity during a typical week and (4) moderate intensity defined as the number of activities of at least moderate intensity (MET value ≥ 3.0) during a typical week.

**Table 4:** Descriptive statistics and reliability coefficients (ICC) of the CHAMPS in French-speaking participants (*N* = 18).

		Visit 1		Visit 2		ICC	95% CI
CHAMPS score^*∗*^		Mean (SD)	Median (IQR^†^)	Mean (SD)	Median (IQR)		
Caloric expenditure^‡^ (kcal/week)	(1) All activities	2061 (1638)	1772 (1610)	1668 (1253)	1189 (1285)	0.70	0.52–0.86
	(2) Moderate-intensity activities	872 (967)	499 (1004)	443 (590)	263 (483)	0.14	−0.13–0.49
Frequency^§^ (times/week)	(3) All activities	15 (7.5)	15.8 (10)	14 (6.1)	13 (10)	0.61	0.39–0.81
	(4) Moderate-intensity activities	5.1 (4.2)	5 (6)	4.2 (3.4)	3.5 (5)	0.68	0.49–0.85

^*∗*^CHAMPS = Community Healthy Activities Model Program for Seniors. ^†^IQR = interquartile range. ^‡^Caloric expenditure scores include (1) all activities defined as the total kilocalories expended during activities of any intensity during a typical week and (2) moderate-intensity defined as the total kilocalories expended during activities of at least moderate intensity (MET value ≥ 3.0) during a typical week. ^§^Frequency scores include (3) all activities defined as the number of activities of any intensity during a typical week and (4) moderate intensity defined as the number of activities of at least moderate intensity (MET value ≥ 3.0) during a typical week.

**Table 5:** Correlations^*∗*^ of CHAMPS caloric expenditure scores with measures of health-related quality of life (HRQoL).

HRQoL measure	Domain	All activities		Moderate-intensity activities	
		English-speaking participants (*N* = 56)^†^	French-speaking participants (*N* = 74)^‡^	English-speaking participants (*N* = 56)^†^	French-speaking participants (*N* = 74)^‡^
SF-36	GH	0.007 (−0.3, 0.3)	0.1 (−0.1, 0.3)	0.3 (0.04, 0.5)^§^	0.2 (−0.03, 0.4)
	RP	0.3 (0.04, 0.5)^§^	0.2 (−0.03, 0.4)	0.2 (−0.07, 0.4)	0.3 (0.08, 0.5)^§^
	PF	0.2 (−0.07, 0.4)	0.2 (−0.03, 0.4)	0.3 (0.04, 0.5)^§^	**0.5 (0.3, 0.7)** ^§^
	BP	−0.2 (−0.4, 0.07)	0.05 (−0.2, 0.3)	0.03 (−0.2, 0.3)	0.05 (−0.2, 0.3)
	MH	−0.02 (−0.3, 0.2)	0.2 (−0.03, 0.4)	0.02 (−0.2, 0.3)	0.3 (0.08, 0.5)^§^
	RE	0.09 (−0.2, 0.3)	0.2 (−0.03, 0.4)	0.1 (−0.2, 0.4)	0.3 (0.08, 0.5)^§^
	SF	0.2 (−0.07, 0.4)	0.2 (−0.03, 0.4)	0.3 (0.04, 0.5)^§^	**0.4 (0.2, 0.6)** ^§^
	VT	0.2 (−0.07, 0.4)	0.04 (−0.2, 0.3)	**0.4 (0.2, 0.6)** ^§^	0.3 (0.08, 0.5)^§^
	PCS	0.2 (−0.07, 0.4)	0.06 (−0.2, 0.3)	**0.4 (0.2, 0.6)** ^§^	0.2 (−0.04, 0.4)
	MCS	0.06 (−0.2, 0.3)	0.2 (−0.04, 0.4)	0.1 (−0.2, 0.4)	0.3 (0.07, 0.5)
SGRQ	Activity	−0.2 (−0.4, 0.07)	0.3 (−0.5, −0.08)^§^	**−0.5 (−0.7, −0.3)** ^§^	**−0.5 (−0.7, −0.3)** ^§^
	Symptoms	0.08 (−0.2, 0.3)	−0.02 (−0.3, 0.2)	−0.1 (−0.4, 0.2)	−0.2 (−0.4, 0.03)
	Impact	0.02 (−0.2, 0.3)	−0.2 (−0.4, 0.03)	−0.3 (−0.5, −0.04)^§^	−0.3 (−0.5, −0.08)^§^
	Total	−0.05 (−0.3, 0.2)	−0.2 (−0.4, 0.03)	**−0.4 (−0.6, −0.2)** ^§^	**−0.4 (−0.6, −0.2)** ^§^

^*∗*^Spearman's Rho and 95% confidence intervals. ^†^English-speaking participants: *N* = 55 SF-36 physical component summary. ^‡^French-speaking participants: *N* = 73 SF-36 physical functioning; *N* = 70 physical component summary. ^§^Statistically significant correlations (95% CI does not include zero; *p* < 0.05). Those in bold met the threshold for moderate correlation (*r* ≥ 0.4).

**Table 6:** Correlations^*∗*^ of CHAMPS frequency scores with measures of health-related quality of life (HRQoL).

HRQoL measure	Domain	All activities		Moderate-intensity activities	
		English-speaking participants (*N* = 56)^†^	French-speaking participants (*N* = 74)^‡^	English-speaking participants (*N* = 56)^†^	French-speaking participants (*N* = 74)^‡^
SF-36	GH	0.3 (0.04, 0.5)^§^	0.1 (−0.1, 0.3)	**0.4 (0.2, 0.6)** ^§^	0.2 (−0.03, 0.4)
	RP	0.3 (0.04, 0.5)^§^	0.05 (−0.2, 0.3)	**0.4 (0.2, 0.6)** ^§^	0.3 (0.08, 0.5)^§^
	PF	0.3 (0.04, 0.5)	0.3 (0.08, 0.5)^§^	0.2 (−0.07, 0.4)	**0.5 (0.3, 0.7)** ^§^
	BP	−0.06 (−0.3, 0.2)	−0.06 (−0.3, 0.2)	0.2 (−0.07, 0.4)	−0.06 (−0.3, 0.2)
	MH	0.08 (−0.2, 0.3)	0.09 (−0.1, 0.3)	0.1 (−0.2, 0.4)	0.2 (−0.03, 0.4)^§^
	RE	0.2 (−0.07, 0.4)	0.06 (−0.2, 0.3)	0.2 (−0.07, 0.4)	0.3 (0.08, 0.5)^§^
	SF	0.3 (0.04, 0.5)^§^	0.1 (−0.1, 0.3)	**0.4 (0.2, 0.6)** ^§^	0.3 (0.07, 0.5)^§^
	VT	0.3 (0.04, 0.5)^§^	0.02 (−0.2, 0.3)	**0.4 (0.2, 0.6)** ^§^	0.3 (0.08, 0.5)^§^
	PCS	0.3 (0.04, 0.5)^§^	0.2 (−0.04, 0.4)	**0.4 (0.2, 0.6)** ^§^	0.2 (−0.04, 0.4)^§^
	MCS	0.2 (−0.07, 0.4)	0.06 (−0.2, 0.3)	0.2 (−0.07, 0.4)	0.3 (0.07, 0.5)^§^
SGRQ	Activity	**−0.4 (−0.6, −0.2)** ^§^	−0.2 (−0.4, 0.03)	**-0.5 (−0.7, −0.3)** ^§^	**−0.4 (−0.6, −0.2)** ^§^
	Symptoms	−0.1 (−0.4, 0.2)	−0.09 (−0.3, 0.1)	−0.2 (−0.4, 0.07)	−0.3 (−0.5, −0.08)
	Impact	−0.2 (−0.4, 0.07)	−0.2 (−0.4, 0.03)	**-0.4 (−0.6, −0.2)** ^§^	**−0.4 (−0.6, −0.2)** ^§^
	Total	−0.3 (−0.5, −0.04)^§^	−0.2 (−0.4, 0.03)	**−0.4 (−0.6, −0.2)** ^§^	**−0.4 (−0.6, −0.2)** ^§^

^*∗*^Spearman's Rho and 95% confidence intervals (*p* < 0.05). ^†^English-speaking participants: *N* = 55 physical component summary. ^‡^French-speaking participants: *N* = 73 physical functioning; *N* = 70 physical component summary. ^§^ Statistically significant correlations (95% CI does not include zero; *p* < 0.05). Those in bold met the threshold for a moderate correlation (*r* ≥ 0.4).

## Data Availability

The quantitative data used to support the findings of this study are included within the article. Data are available on request by contacting susanne.mak@mcgill.ca.
